# Changes in the Upper Airway Dimension Following the Use of Functional Appliances in Children with Obstructive Sleep Apnea: A Systematic Review

**DOI:** 10.3390/children12020227

**Published:** 2025-02-13

**Authors:** Andrea Scribante, Maurizio Pascadopoli, Paolo Zampetti, Chiara Rocchi, Francesca Falsarone, Maria Francesca Sfondrini

**Affiliations:** Unit of Orthodontics and Pediatric Dentistry, Section of Dentistry, Department of Clinical, Surgical, Diagnostic and Pediatric Sciences, University of Pavia, 27100 Pavia, Italy; maurizio.pascadopoli01@universitadipavia.it (M.P.); chiara.rocchi01@universitadipavia.it (C.R.); francesca.falsarone01@universitadipavia.it (F.F.); francesca.sfondrini@unipv.it (M.F.S.)

**Keywords:** orthodontic appliances, sleep apnea syndromes, sleep disorders, respiration disorders

## Abstract

**Introduction**: Obstructive Sleep Apnea Syndrome (OSAS) is a sleep-related breathing disorder common in children, often linked to craniofacial anomalies like retrognathic mandibles in Class II malocclusions. Functional appliances (FAs) have been proposed as non-invasive treatments to improve OSAS symptoms by modifying upper airway dimensions. Objective: this systematic review evaluates the effectiveness of functional appliances in improving upper airway structures in children with OSAS. **Materials and Methods**: the study was conducted according to PRISMA guidelines, analyzing studies published between 2004 and 2024 on PubMed, Scopus, Web of Science, Embase, and Cochrane Library databases. Inclusion criteria focused on growing patients (≤14 years) with OSAS and Class II skeletal malocclusions treated with FAs. **Results**: Of 1298 articles screened, four studies met the inclusion criteria. All studies reported a significant increase in upper airway dimensions of approximately 21% after treatment with FAs. Specifically, the cephalometric and tomographic evaluations revealed a clear enlargement of the superior posterior airway space of about 0.5 mm and a consequent improvement of the respiratory function. Discrepancies arose regarding changes in soft palate length and hyoid bone position, perhaps due to the measurement methods’ variation. **Conclusions**: Functional appliances appear effective in improving upper airway dimensions and alleviating OSAS symptoms in children. However, the limited number of studies, small sample sizes, and short follow-up periods emphasize the need for further research to confirm long-term efficacy and standardize evaluation protocols.

## 1. Introduction

Obstructive Sleep Apnea Syndrome (OSAS) is disordered breathing in sleep characterized by intermittent, partial, or complete collapse of the upper airway [1] that determines an abnormal sleep rhythm [2]. This syndrome has a prevalence of 3% in children and 3–6 years old patients are particularly affected [2,3]. The etiology is multifactorial and each genetic or functional factor that contributes to the collapse or obstruction of the upper airway is considered a potential risk factor [4]. The main risk factors are adenotonsillar hypertrophy and obesity followed by altered muscular function, asthma, and syndromic injuries [5]; among craniofacial disorders, Class II malocclusions due to retrognathic mandibles can be found [6]. Severe mandibular retrognathism has been associated with OSAS [7], as the posterior positioning of the tongue and hyoid bone can contribute to a simultaneous narrowing of the upper airway [8], increasing the likelihood of respiratory issues during the day and nocturnal disturbances [9]. Therefore, functional appliances can improve the symptomatology of OSAS in children, reducing airway collapse [10].

The upper airway is formed by the external nose, nasal cavity, paranasal sinuses, and nasopharynx. It is divided into three regions: the nasopharyngeal airway—from the top of the nasopharynx to the plane of the hard palate; the oropharyngeal airway—from the plane of hard palate to the top of the epiglottis, and the laryngopharyngeal airway—from the top to the bottom of the epiglottis [11]. The assessment of the upper airway is conducted using either Cone Beam Computed Tomography (CBCT) or lateral cephalometric radiography. These imaging techniques enable the identification of key anatomical landmarks, facilitating the interpretation of this structure in three or two dimensions, respectively. Recent studies utilizing X-ray imaging have highlighted the relationship between craniofacial growth, functional aspects, and the impact of orthodontic treatment on the three-dimensional configuration of the airway [12].

Several studies have been conducted in recent years to determine changes in the upper airway of healthy patients with Class II mandibular retrognathism treated with functional mandibular advancement devices (MADs) [13,14,15]. However, current findings are inconsistent and often contradictory. Specifically, several reviews have emphasized the positive effects of functional appliances’ treatment on the AHI for the upper airways in growing individuals with Class II malocclusion [16,17]. While some studies have explored the effectiveness of functional appliances in treating OSAS [18,19], there is a considerable lack of evidence regarding the upper airway changes in patients with sleep disorders. In addition, according to the American Academy of Pediatric guidelines, the primary treatment for patients with OSA is adenotonsillectomy. Consequently, therapy with functional appliances can help improve the symptomatology of the syndrome but not resolve it [20,21]. The need for the present systematic review arises from the presence of multiple limitations in the literature regarding the effectiveness of FAs. In particular, among these, imprecise description and assessment of the sample’s age, variability in the age range, absence of a uniform baseline orthodontic diagnosis, lack of description of the appliance used, and insufficient detail regarding the activation protocol are noted. Furthermore, in the various studies, therapy with FAs was conducted not with the aim of improving OSA, but rather to correct dentoskeletal issues [22]. In conclusion, the aim of this systematic review is to analyze the current literature to evaluate the effectiveness of FAs in resolving or improving OSA.

## 2. Materials and Methods

### 2.1. Search Strategy

This systematic review was performed in accordance with the Preferred Reporting Items for Systematic Reviews and Meta-Analyses (PRISMA) guidelines [23].

The review protocol was not registered prior to the study.

The initial study search was performed on Pubmed, Scopus, Web of Science, Cochrane Library, and Embase online databases for the period of 2004 to 2024 (last screening on 18 November 2024). The search query was the following: (sleep disorders OR obstructive sleep apnea OR sleep-related breathing disorders OR sleep disordered breathing) AND (orthodontics OR orthodontic treatment OR functional appliance) AND (children OR pediatric OR pediatrics) AND (upper airway OR upper airway changes). After removing duplicates, a study design filter was applied based on the inclusion and exclusion criteria outlined in Table 1. All the retrieved studies were screened by titles and abstracts, by two authors independently (F.F. and C.R.). The studies selected for full-text reading were independently screened by two authors (F.F. and C.R.) according to the inclusion/exclusion criteria reported in Table 1. Eventual controversies were solved by the intervention of the senior author (M.P.).

**Figure 1:**
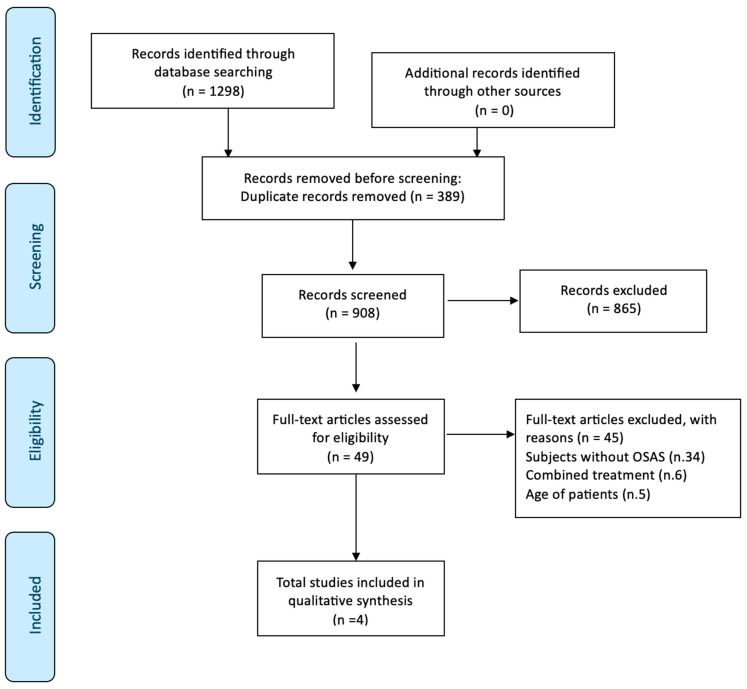
PRISMA flow chart.

The PRISMA checklist was compiled (Supplementary Materials). The search flow diagram reported in Figure 1 summarizes the search strategy.

To reduce potential bias related to language selection, all non-English papers were included in the title and abstract screening phase and screened if their abstracts were available in English.

### 2.2. Selection Criteria

The PICOS criteria were used to determine whether a study should be included or excluded:

- Participants: growing patients (14 years old and younger) diagnosed with OSAS and skeletal class II malocclusion due to retrognathic mandible, without craniofacial syndromes.
- Intervention: interceptive orthopedic treatment with functional appliances only.
- Comparison: with or without a control group or pre-treatment and post-treatment.
- Outcome: upper airway modifications
- Study design: randomized/non-randomized clinical trials, cohort studies, cross-sectional studies, case–control studies, prospective studies, retrospective studies, pilot studies.

Studies were excluded if the sample included patients over 14 years of age, patients without OSAS, or those undergoing combined orthodontic treatments. Furthermore, studies lacking pre- and post-treatment airway measurements obtained through CBCT or cephalometric analysis were not considered.

### 2.3. Data Extraction

The following information was retrieved from included studies: authors, publication date, study design, setting, patient characteristics, interventions, number of patients, genders, age, treatment/observation duration and outcomes, image examination.

## 3. Results

The search revealed 1298 total results. After removing the duplicates and checking for the presence of keywords in the title and abstract, a total of 49 articles were selected for full-text reading. Further screening excluded 45 articles for the following reasons: subjects without OSA (34), combined treatments, those that use FAs combined with other therapy (6), and the age of patients (4). Four articles were included for data extraction (Table 2). Overall, 191 patients were involved in the included studies, with a mean age of 9.6 years. In particular, excluding the study by Zreqat [24], the percentage of males is equal to 59% (mean age 10.5) and the percentage of females is equal to 41% (mean age 10.7). Regarding the study population, Zreqat [24] evaluated a homogeneous sample both in terms of the number of tests and controls as well as the mean age. Pavoni [25] used a control group with a larger number compared to the tests (51/31), while the mean age remained homogeneous. It can be stated that this is the same as the study by Maspero [9], which presents a number of tests quadruple to that of the controls. A significant difference between the number of controls and tests may lead to a reduced validity of the obtained results.

Regarding the morphology of the airways, all studies included in the review have shown that after treatment with functional appliances, the airway space significantly increased.

**Figure 2:**
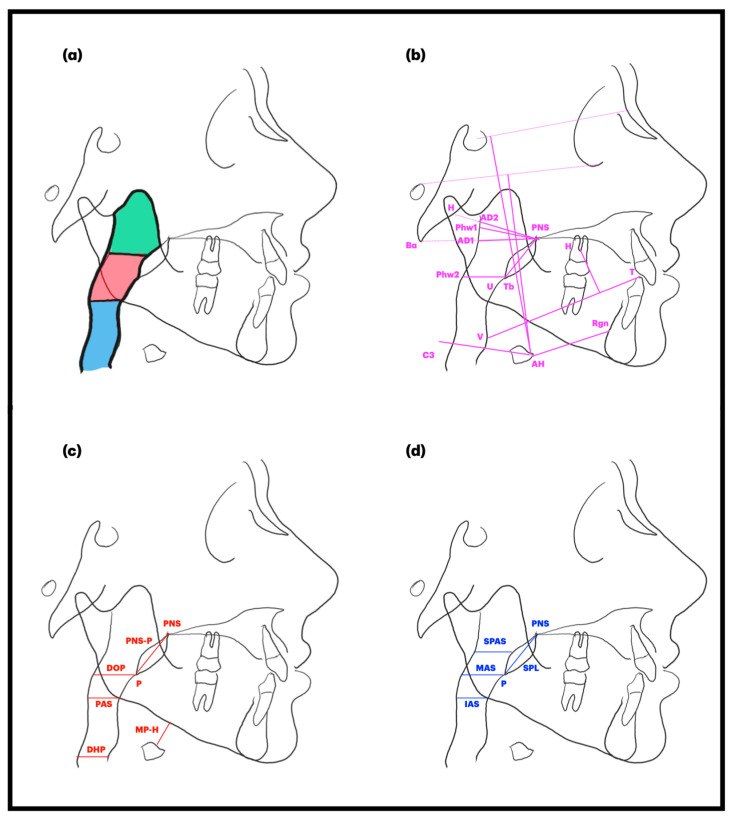
Airway analysis from selected studies in review according to (**a**) Zreaqat et al. [23] (volume and MCA of three different areas: nasopharynx in green, oropharynx in red and hypopharynx in blue); (**b**) Pavoni et al. [24]; (**c**) Maspero et al. [9]; (**d**) Zhang et al. [25].

The prospective study by Zreaqat et al. [24] found that nine months of twin-block therapy significantly improved upper airway parameters at the oropharynx level compared to controls. The therapy increased volume by 3137 mm^3^ and the MCA of the oropharynx in the axial view by 40.47 mm^2^. In the hypopharynx, only the MCA showed a significant increase (19.91 mm^2^). Pavoni et al. [25] reported that there is a significant increase in the superior posterior airway dimension (Phw1-Psp, +0.5 mm) and in the lower airway dimension (Phw2-Tb, +0.6 mm). Significant increases were also observed in pharyngeal width (MPW, +2.1 mm), upper airway dimension (PNS-AD1, +1.2 mm), and in lower airway dimension (PNS-AD2, +1.2 mm), along with a reduction in adenoid thickness in upper airways (AD2-H, −1.0 mm) and in adenoid thickness in the lower airways (AD1-Ba, −0.4 mm). Maspero et al. [9] evaluated the Andresen appliance before and after 16 months of treatment and the therapy significantly increased DOP (+2.43 mm), lower pharyngeal space (DPH, +2.09 mm), and posterior airway space (PAS, +0.74 mm). Zhang et al. [26], after twin-block therapy, found a 75.9% reduction in AHI, and an increase in superior posterior airway space (SPAS, +3.79 mm) and middle airway space (MAS, +5.87 mm). Moreover, Pavoni et al. [25], Maspero et al. [9], and Zhang et al. [26] found controversial results for soft palate length (SPL). Pavoni [25] and Maspero [9] found an increase in SPL (+1.66 mm/+3.95 mm) after treatment with a modified monoblock and Andersen appliance, respectively, whereas Zhang [26] found a decrease in SPL (−5.93 mm) following treatment with a twin-block. The discrepancy in the measurement of SPL may be attributed to the different reference points considered by the authors. Specifically, Maspero and Pavoni measure SPL as the distance between the tip of the uvula and PNS (U-PNS). In contrast, Zhang evaluates SPL as the distance between the tip of the soft palate and PNS (P-PNS). Furthermore, Maspero et al. [9] pointed out that hyoid position was superior (MP-H, −4.93 mm) after treatment with the Andersen appliance, while Pavoni et al. [25] found that the hyoid bone moved more anteriorly (AH-C3 horizontal: +3.6 mm) and in a lower position (AH-SN, +7.2 mm; AH-FH, +6.4 mm). Figure 2 presents the points that have been analyzed in the included studies.

### Risk of Bias

Table 3 provides an overview of the risk of bias for the articles analyzed in this review. Overall, this review demonstrates a relatively moderate risk of bias. The greatest risk of bias is associated with the adequacy of the sequence generation, while a moderate risk is related to blinding. In contrast, the results show a low risk of bias.

The risk of bias for each study was assessed using the Cochrane Risk of Bias Tool (RoB 2) [27], which evaluates five key domains: sequence generation, allocation concealment, blinding, incomplete outcome data, and selective reporting of outcomes. Each domain was classified as having a high, low, or unclear risk of bias.

The adequacy of sequence generation was examined, with studies assigned a high risk if the randomization process was unclear or inadequate. Allocation concealment was assessed based on whether the allocation sequence was properly concealed from researchers and participants. A high risk was given if this process was not adequately concealed. The presence of blinding was also considered, with moderate risk assigned to studies lacking clear blinding procedures. The handling of incomplete outcome data was evaluated, and studies with significant missing data or improper handling were assigned a higher risk. Finally, selective reporting of outcomes was assessed, with a high risk given to studies that did not register or report all pre-specified outcomes accurately.

Table 3 provides a visual summary of the risk of bias for each study using color-coded icons: green represents no risk of bias, yellow indicates moderate risk, and red indicates high risk.

## 4. Discussion

The treatment of OSAS in children should focus on risk factors such as obesity and craniofacial abnormalities to resolve airway obstruction [28]. Some children do not respond to standard treatments, such as adenotonsillectomy, or cannot tolerate CPAP. Functional orthodontic appliances are a less invasive solution, stimulating mandibular growth and improving the dimensions of the upper airways [12]. Recently, the pharyngeal airway has garnered significant interest among orthodontists, not only because of its connection to respiratory function and craniofacial growth and development, but also due to the impact of orthodontic treatment on constricted airway dimensions. OSAS is associated with deviations in craniofacial growth, such as maxillary constriction and Class II skeletal type, which are known risk factors for OSAS [29]. The relationship between these craniofacial morphologies and the development of OSA is not fully established. However, some studies have identified three phenotypes of OSA in adults, one of which, the skeletal type, would be most suitable for orthopedic or surgical modifications of the craniofacial structure [30]. Research indicates that FAs may offer a promising treatment strategy for enhancing airway dimensions in growing patients with skeletal Class II malocclusion [14,31,32]. The effect of FAs on airway dimensions remains a matter of debate. Additionally, as previously highlighted, few studies focus on airway changes as the primary outcome in patients with OSAS undergoing FA treatment. To fill this gap, the present systematic review was conducted to investigate the impact of FAs on upper airway dimensions in growing patients with skeletal Class II malocclusion and OSAS. FAs are used to correct skeletal Class II malocclusion through skeletal and dentoalveolar changes, although their primary mechanism remains debated. After the correction of Class II malocclusion, the forward positioning of the mandible and the hyoid bone resulted in an anterior pull on the tongue, which led to an increase in the posterior airway space and a decrease in airway resistance [33]. This leads to an increase in oxygen saturation, improves OSA symptoms, and consequently enhances quality of life, behavior, and school performance.

While earlier studies suggested FAs can stimulate mandibular growth and anterior repositioning [12,34], recent systematic reviews indicate minimal skeletal changes, with dentoalveolar adjustments playing a more significant role [35,36]. Although there are no significant changes in mandibular length, an increase in the SNB angle was observed, improving the oropharyngeal space. Additionally, the hyoid bone shifts forward, but this effect was also found in the control group, suggesting that growth plays a more important role than treatment. Dentoalveolar effects, such as the mesial movement of lower teeth, may indirectly expand the pharyngeal airway because they could pull the tongue and hyoid bone forward, causing adjustments in the soft palate and leading to an increase in the dimensions of the pharyngeal airway [12,37]. The studies analyzed in this review emphasized changes in various airway parameters among patients with OSA, as identified through polysomnography (PSG). The parameters for assessing airway changes following treatment with (FAs) were obtained by subjecting patients to CBCT or cephalometry, both before and after treatment.

Regarding the upper airways, all studies have shown a significant increase in their dimensions following treatment with FAs. However, the effectiveness of a mandibular advancement appliance for treating obstructive sleep disorders remains a topic of debate, with varying success rates reported in clinical studies [38]. This variability may be attributed to differences in research protocols, the design of the appliances used, and the selection of subjects. In particular, the study by Zreaqat [24] demonstrated that upper airway parameters at the level of the oropharynx improve significantly in volume and MCA in the axial view and at the level of hypopharynx only MCA increased significantly with twin-block therapy compared to controls. Transverse dimensional changes were more pronounced than sagittal ones, consistent with clinical evidence. So, MCA stands out as a key parameter for understanding the anatomical role of the airway in pediatric OSA pathogenesis. These findings align with the study by Pavoni et al. [25], which also observed a significant increase in the dimension of upper and lower airways in the treatment group, with an increase in superior posterior and inferior airway space. Additionally, the middle pharyngeal width and upper and lower airway thickness showed a significant increase, and they also found a reduction in upper and lower adenoid thickness. Similar results were reported in the studies by Zhang et al. [26] and Maspero et al. [9]: in the former study, an increase in superior posterior and middle airway space was found; in the latter study, it was found that the middle pharyngeal space, the lower pharyngeal space, and the posterior airway space increased significantly. Therefore, regarding airway modifications, the studies by Zreqat, Pavoni, Maspero, and Zhang agree in affirming that treatment with twin-block, monoblock, or Andresen appliances results in an increase in the middle and superior posterior airway space. It is important to highlight that the measurements performed in the various studies are not standardized, which does not allow the quantitative synthesis of data. The same evidence has been demonstrated in additional studies that treated patients with skeletal Class II malocclusions using functional mandibular advancement appliances, but without considering the presence of OSAS. For example, in the study of [14], Bionator and Activator appliances were used for treating Class II malocclusions, with an increase in upper airway dimensions compared to controls during treatment and in the long term. The effectiveness of twin-block therapy was also evaluated in the case–control study by Ali et al. [32], which highlighted an increase in airway dimensions during treatment. This finding was also reported in studies by Yildirim [39] and Thakur [40].

Most likely, effects on upper airways are due to the forward traction of the tongue following the anterior repositioning of the mandible and the hyoid bone, as demonstrated in the study of Lione et al. [41]. These results lead to an increase in posterior airway space and a reduction in resistance, thereby improving breathing, as also stated by Schütz in his study [33]. In this regard, Pavoni [25] reported a direct beneficial effect, with an anterior repositioning of the tongue (V-T, +5.7 mm); this is the result of the hyoid bone moving more anteriorly and in a lower position. By contrast, another study demonstrated that the hyoid bone moved anteriorly but not vertically [27] and another one found that the hyoid bone moves superiorly after treatment with an activator [9]. In addition, the study of Battagel [42] demonstrated that the hyoid bone moved upwards and backwards in relation to the mandibular plane following mandibular protrusion. In conclusion, the effects on the hyoid bone following treatment with FAs are varied and unclear; this may be due to the lack of standardized measurements for assessing changes in the hyoid.

Another variable in which differing values were observed in the analyzed studies was the length of the soft palate. Pavoni [25] and Maspero [9] reported an increase in the soft palate length after treatment with a modified monoblock and Andersen appliance, respectively. In contrast, Zhang [26] observed a reduction in soft palate length following treatment with a twin-block.

Orthodontic interventions utilizing oral appliances are viewed as a promising supplementary treatment option for SDB in children. Orthodontists are increasingly recognized for their vital role in addressing snoring and respiratory issues through the use of oral mandibular advancement devices and rapid maxillary expansion. On the other hand, the small and non-homogeneous sample size, the variability in measurement methods, the fact that only two studies have quantified the effect of OSA, the short evaluation period, and the absence of patient monitoring following therapy reduce the generalizability of the findings. Therefore, despite all studies yielding positive results, it is important to emphasize that each has utilized different and inconsistent parameters to assess the effectiveness of FAs. This may be due to the absence of standardized measures for evaluating airway conditions, which would enhance the validity of the result.

## 5. Conclusions

In conclusion, functional appliance therapy appears to lead to modifications in airway dimensions. The findings of the studies included in the review suggest functional appliances may be a promising adjunct therapy for improving upper airway dimensions in children with OSAS, but further studies with larger sample sizes and standardized protocols are needed.

## Figures and Tables

**Table 1:** Inclusion/exclusion criteria.

	**Inclusion Criteria**
Type of study	Randomized/non-randomized trials, cohort studies, cross-sectional studies, case–control studies, prospective studies, retrospective studies, pilot studies
Cohort	Growing patients (14 years old and younger) with OSAS and skeletal class II malocclusion due to retrognathic mandible, treated with functional appliance
Data	Evaluation of upper airway modification through cephalogram or CBCT
Timing	Pre- and post-treatments
	**Exclusion Criteria**
Type of study	Case series, case reports, reviews, letters, technical notes, conference, documents, books, book chapters, editorials, surveys
Cohort	Studies on patients with >14 years old, without OSAS, treated with combined treatment. Studies on craniofacial syndromic patients
Data	Evaluation of efficacy of functional treatment without upper airway measures on cephalograms or CBCT
Timing	Only pre- or post-treatments

**Table 2:** Characteristics of the studies included in the review.

Study	Study Design	Setting	Characteristics of Patients	Interventions	No. of Patients (M/F)	Age in Years	Treatment/Observation Time	Imaging Examination	Outcomes
Zreaqat 2023 [24]	Prospective longitudinal study	Malaysia	Class II malocclusion, 79° < SNA < 84°, SNB > 76°, 20° < FMA < 28°, 6 < OJ < 10, GT: OSA GC	GT: twin-block GC: prefunctional therapy	GT: 34 GC: 34	GT: 8–12 GC: 8–12	GT: 9 m	CBCT	Compared to controls, treatment group showed an increase in the volume of the airway and in the MCA hypopharynx. Furthermore, after treatment, AHI dropped significantly.
Pavoni 2017 [25]	Prospective case–control study	Rome	GT: Class II malocclusion (ANB > 4, OJ > 5) and SDB GC: Class II malocclusion	GT: modified monoblock	GT: 51 (27/24) GC: 31 (15/16)	GT: 9.9 GC: 10.1	GT: 1.8 y GC: 1.9 y	Cephalogram	Compared to controls, the treatment group showed an increase in SPL, the posterior inferior airway space, and in the middle pharyngeal width. Moreover, it was registered as an anterior position of the hyoid bone, an anterior and lower position of the tongue, and a reduction in the OSA’s symptoms.
Maspero 2015 [9]	Prospective study	Milan	GT: class II malocclusion (SNA = 82 ± 2), SNB < 80 and OSA, GC	GT: Andresen appliance	GT: 40 (23/17) GC: 10 (5/5)	GT: M: 10/14 F: 8/10 GC: M: 11/14 F: 9/10	GT: 16 m	CBCT	Compared to controls, the treatment group showed an increase in DOP, PAS, and SPL. Moreover, the hyoid moved in a superior manner and breathing parameters were better after treatment.
Zhang 2013 [26]	Preliminary study	Wuhan	GT: ANB > 3, SNB < 80°, OJ > 3 and OSA	GT: twin-block	GT: 46 (31/15)	GT: 9.7 ± 1.5	GT: 10.8 m	Cephalogram	After the treatment, the AHI dropped about 75.9%, the superior posterior and middle airway space increased (SPAS, MAS). Moreover, a reduction in SPL was pointed out.

GT: treatment group; GC: control group; FMA: Frankfurt mandibular plane angle; OJ: overjet; y: years; m: months; MCA: minimal cross-sectional area; AHI: apnea–hypopnea index; OSA: Obstructive Sleep Apnea; SDB: sleep disordered breathing; SPL: soft palate length; CBCT: cone beam computed technology; DOP: U-MPW; PAS: posterior airway space; SPAS: superior posterior airway space; MAS: middle airway space; SNA: angle between the sella, nasion and A point; SNB: angle between the sella, nasion and B point; ANB: angle between point A, the nasion and point B.

**Table 3:** Risk of bias. The colored faces indicate the level of risk: red (high risk), yellow (unclear risk), and green (low risk).

	Adequate Sequence Generated	Allocating Concealment	Blinding	Incomplete Outcome Data	Registration Outcome Data
Zreaqat 2023 [24]	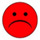	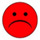	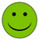	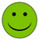	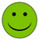
Pavoni 2017 [25]	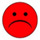	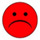	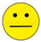	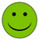	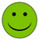
Maspero 2015 [9]	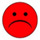	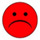	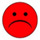	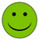	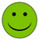
Zhang 2013 [26]	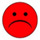	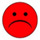	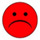	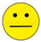	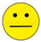

## Supplementary Materials

The following supporting information can be downloaded at www.mdpi.com/article/10.3390/children12020227/s1, PRISMA checklist. Ref. [43] is cited in Supplementary Materials.

## Abbreviations

The following abbreviations are used in this manuscript:

OSAS	Obstructive Sleep Apnea
CBCT	Cone beam computed tomography
FA	Functional appliance
MCA	Minimal cross-sectional area
Phw1-psp	Upper airway dimension
Phw2-tb	Lower airway dimension
MPW	Pharyngeal width
PNS-AD1	Upper airway dimension
PNS-AD2	Lower airway dimension
AD2-H	Adenoid thickness in upper airway
AD1-Ba	Adenoid thickness in lower airway
DOP	U-MPW
DPH	Lower pharyngeal space
PAS	Posterior airway space
AHI	Apnea–Hypopnea index
SPAS	Superior posterior airway space
MAS	Middle airway space
SPL	Soft palate length
SNA	Angle between the sella, nasion and A point
SNB	Angle between the sella, nasion and B point
ANB	Angle between point A, the nasion and point B
FMA	Frankfurt mandibular plane angle
OJ	Overjet
SDB	Sleep disordered breathing
CPAP	Continuous positive airway pressure
PSG	Polysomnography
